# Comparison of Two Simulated Procedural Assessment Formats in Attending Emergency Physicians

**DOI:** 10.7759/cureus.14943

**Published:** 2021-05-10

**Authors:** Sara M Hock, Shital C Shah, Priya D Perumalsamy, Michelle Sergel

**Affiliations:** 1 Department of Emergency Medicine, Rush University Medical Center, Chicago, USA; 2 Department of Health Systems Management, College of Health Sciences, Rush University Medical Center, Chicago, USA; 3 Department of Emergency Medicine, Cape Regional Medical Center, Cape May Court House, USA; 4 Department of Emergency Medicine, Cook County Health and Hospital System, Chicago, USA

**Keywords:** simulated assessment, continuing medical education, emergency medicine, procedural training, oppe

## Abstract

Background

Emergency physicians must be proficient at inserting central venous catheters and performing lumbar punctures to provide life-saving therapies to critically ill patients. An assessment of procedural skill is rarely performed after an emergency physician has completed residency. Current board certification exams for emergency medicine focus only on verbal descriptions of procedures to assess skill. We compared two methods of procedural skill assessment, simulated task trainer and verbal description, to assess the range of skill in central venous catheter insertion and lumbar punctures of emergency attending physicians at a large, urban, academic tertiary care institution.

Methodology

This is a prospective cohort study of simulated internal jugular central venous catheter insertion and lumbar puncture skill by emergency attending physicians on a task trainer versus verbal description. A total of 17 attending emergency medicine physicians consented to participate in the study during a yearly procedural skills session. For each subject, two expert raters used previously published checklists to assess procedural skill and give a global rating score.

Results

More checklist items were performed correctly on the task trainer than on verbal assessment for central line (task trainer = 78.4% ± 8.32% and verbal = 68.26% ± 8.9%) and lumbar puncture (task trainer = 85.57% ± 7.6% and verbal = 73.53%4 ± 10.34%) procedures, both with significant differences (p < 0.001). Of the participants, 82% strongly preferred the task trainer format to the verbal description assessment format.

Conclusions

The higher scores on the simulated format compared to the current verbal format imply that a shift towards simulated procedural assessment techniques may benefit examinees. More work is needed to determine if objective checklist scores for practicing attending emergency physicians correlate with subjective expert assessments of their procedural skills.

## Introduction

Since 2007, the Joint Commission on Accreditation of Healthcare Organizations has required evaluation of practicing attending physicians in focused and ongoing provider performance evaluation (OPPE). This global assessment is intended to be an evaluation of a practitioner’s clinical competence, and as such could reasonably include a direct observation of procedural skill. One study reporting current practices in emergency medicine (EM) did not report clinical or simulated procedural assessment as a component of current OPPE practice [1]. Such assessment may be an important component as procedural skills decay over time without practice [2]. There are several methods by which this can be accomplished, including through chart review, direct observation of patient care, or by verbally assessing knowledge of the steps required to do a particular procedure [3]. However, for infrequently performed or complicated procedures, these methods may not allow objective evaluation on a regular basis. Simulation using task trainers offers an alternative method of objective evaluation of procedures in a standardized setting.

Simulation-based medical education (SBME) with deliberate practice has been shown to be superior to traditional clinical education with respect to procedural skills in physicians in training, with an overall effect size of 0.71 favoring simulation training with deliberate practice in a meta-analysis [4]. Research supports the use of simulation in resident education due to increased skill following training and improved patient safety [4-7]. The optimal interval for procedural training has not been determined, but studies with residents suggest that training in central venous catheter insertion is retained over at least one year [8]. A study of complex skill retention in attending anesthesiologists showed that cricothyrotomy skills are retained for at least a year following a single case training session [9]. Transfer of skills from simulation to clinical practice has been studied among anesthesiologists and has been shown to result in better performance in a clinical environment [10,11].

Assessment techniques involving simulation such as the objective structured clinical evaluation (OSCE) have been used in undergraduate medical education and are well supported by literature [12-14]. Procedural OSCEs in graduate medical education have been developed and tested, showing higher scores in senior internal medicine residents, an increase during the course of residency, and correlation with national high-stakes examination performance [15-17]. The advantages of such experiences include reproducibility, patient safety, standardization, and the opportunity for several repeated measures to compose an overall assessment.

When used in continuing medical education (CME) for attending physicians, simulation has been shown to be feasible and to increase clinician confidence in patient management [18]. A study of nephrology fellows and attendings showed that both groups scored similarly on a pre-test examination of temporary dialysis catheter placement on task trainers, and that all fellows who completed mastery learning subsequently passed the checklist assessment at the competency level and rated the training session highly [19]. Conversely, Barsuk et al. found that attending physicians across a range of specialties performed inconsistently and missed critical steps when assessed on a central line checklist utilizing a task trainer [20].

Several specialties now require or suggest simulation during residency training or continuing certification. In psychiatry, simulation has been used for OPPE assessments by utilizing online simulated patients and assessments as a way of identifying a smaller number of attendings requiring focused assessment in person [21]. The American Board of Anesthesiology incorporated a simulation component into their maintenance of certification process which is now optional [22]. Surgical residents graduating after 2017-2018 are now required to take and pass the skills portion of the flexible endoscopic surgery curriculum [23], which is one of the first skills assessments used as a prerequisite to board certification. Multiple procedural checklists have been developed but these are primarily for resident training and initial competency assessment [5,6,19,24,25]. In comparison, the current practice in emergency medicine initial certification includes only a verbal description of procedures as part of the oral board examination [3].

Unlike residents in training or newly board-certified physicians, attending emergency physicians are at a variety of experience levels. Skill attrition occurs [2], and procedural frequency varies by patient population and acuity. This group would therefore be expected to have a wide range of skill level. The ability of simulation to present a standardized, specific procedural task offers an opportunity to efficiently assess attending emergency physicians on a variety of critical procedures while simultaneously providing an opportunity of periodic practice. Verbal description, as is currently used in the American Board of Emergency Medicine (ABEM) initial certification examination, was identified as a control for this study as it has been validated for this use by the ABEM. However, verbal description does not assess the psychomotor skill required to perform the procedure, nor does it provide the kinesthetic cues that may trigger performance of subsequent steps. We hypothesized that there would be significant differences between the scores obtained on a verbal procedural assessment (control) and the simulated task trainer assessment of lumbar puncture and central line insertion. This article was previously presented as a meeting abstract at the ACEP Annual Meeting on October 18, 2016 and the International Meeting on Simulation in Healthcare on January 29, 2017.

## Materials and methods

This is an observational cohort study comparing two methods of procedural proficiency assessment in a cohort of practicing attending emergency medicine physicians at a large 671 bed academic medical center (AMC). The Emergency Department (ED) at the AMC consists of 60 beds with an annual census of over 70,000 visits. The Institutional Review Board approved the study and subjects provided their written informed consent before participation.

The ED is staffed by board-certified or board-eligible emergency medicine physicians, all of whom are credentialed to perform a variety of procedures. Our yearly OPPE includes procedures assessed in the simulation laboratory using task trainers. Two simulated scenarios were developed to assess skill in placing central line catheters and in performing a lumbar puncture. Subjects who consented were also surveyed on their attitudes of this simulated format. Each participant completed both procedures using a simulation (task trainer) and verbal assessment technique, the order of which was randomly assigned by random number generator.

Demographic data were obtained from participants, including gender, number of years in practice, and an estimation of the number of central lines and lumbar punctures performed in the past year. Two raters utilized previously validated checklists [5,6] containing 29 items for central line placement and 21 items for lumbar puncture placement. Raters were attending emergency physicians with experience in simulation, and all were trained using an example evaluation to ensure their rating was accurate on each checklist. There were eight raters overall. Raters also provided a global assessment on a Likert scale ranging 1 to 5, with 5 representing the highest score, of their confidence that the physician could perform the procedure proficiently. The purpose of the global assessment was to allow comparison of objective and subjective data regarding procedural skill. For each participant, one rater observed and rated the procedure in real-time and one rater performed a delayed assessment of the same checklist by video recording. All participants completed surveys regarding their attitudes of simulation as a learning experience, as an assessment tool, and rated the likelihood that they would complete a future simulation session. Finally, 14 participants completed a retrospective assessment reporting the number of procedures they had performed in the past year, as well as their confidence in their skills on the procedures before and after the simulation experience.

Most of the checklist items (observed versus not observed) were completed by the raters. The few checklist items that were omitted by raters were coded as not observed. There were three subjects for whom the second rater data was not available due to loss of video recordings. To maintain the sample size, the second ratings were assumed to be similar to the first ratings. The interrater reliability for the two procedures and two assessment formats was measured using Cohen’s kappa. The overall score per subject per procedure was calculated by adding both raters’ observed items and dividing the total by two. In addition, the percentage of checklist items observed was calculated by dividing the overall score by the total number of items in the checklist. An expert panel had determined the minimum passing score (MPS) for these checklists to be three or less missed items [5,6]. Thus, the passing threshold for central line placement was 89.7% (26/29) and for lumbar puncture was 85.7% (18/21). The global assessment for each subject was determined by averaging the two raters’ global assessments.

The impact of the assessment format on average number of checklist items observed, percentage checklist items observed, and overall global assessment was analyzed using paired t-test as well as mixed-model, repeated-measured analysis of variance. The impact of the assessment format on procedure competency was analyzed using Chi-square test. The descriptive analysis for the survey questions used frequencies. In addition, a correlation analysis between percentage checklist items observed and overall global assessment was conducted. Independent sample t-test was utilized to investigate the relationship between procedure competency and overall global assessment. To conduct the analysis, we utilized Microsoft Excel as well as SPSS statistical software (IBM Corp., Armonk, NY).

## Results

All 26 emergency medicine attending physicians completed the yearly OPPE process, and 17 consented to participate in the study protocol. Table 1 demonstrates the characteristics of the study group.

**Table 1:** Characteristics of the study group. SD: standard deviation; IQR: interquartile range

Variables (n = 17)		Frequency/Median (IQR)
Gender	Male	9 (52.9%)
	Female	8 (47.1%)
Years in practice	<5 years	7 (41.2%)
	5-10 years	5 (29.4%)
	>10 years	5 (29.4%)
Number of central lines procedures performed in last 1 year		2.5 (1-5)
Number of lumbar puncture procedures performed in last 1 year		4 (2-7.5)
Pretest confidence in placing central line – Likert (±SD)		4.7 (±0.5)
Pretest confidence in performing lumbar puncture – Likert (±SD)		4.5 (±0.6)

The interrater reliability between raters for the 29-item central line checklist for 13 subjects was moderate with average kappa values of 0.47 and 0.53 for task trainer and verbal assessment techniques, respectively. Similarly, the interrater reliability for the 21-item lumber puncture checklist was also moderate with average kappa values of 0.47 and 0.41 on task trainer and verbal assessment techniques, respectively.

**Figure 1:**
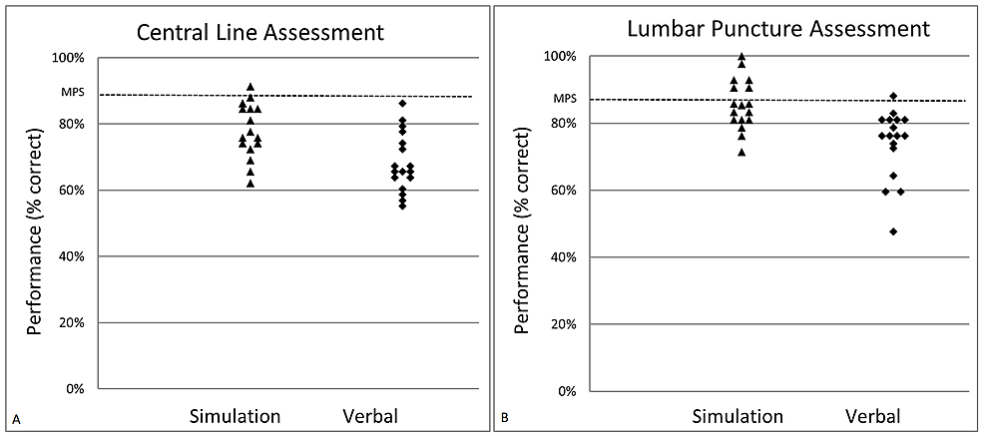
Graphic representation of central line (A) and lumbar puncture (B) performance. Scores are for each subject (n = 17) on simulation and verbal assessment. MPS: minimum passing standard

The average percentages of checklist items completed for central line were 78.4% ± 8.3% and 68.3% ± 8.9% for task trainer and verbal assessment techniques, respectively, a statistically significant difference (t = 6.3, df = 16, p < 0.001). The average percentages of checklist items completed for lumbar puncture were 85.6% ± 7.6% and 73.5% ± 10.3% for task trainer and verbal assessment techniques, respectively (t = 6.75, df = 16, p < 0.001). Graphic representations of checklist scores are presented in Figures 1A, 1B. Few subjects achieved the MPS of three or less missed items on either procedural trainer (6% on central line task trainer and 35% on lumbar puncture task trainer), and only one subject achieved the MPS in the verbal format. The overall global rating scores for central line were 4.8 ± 0.4 and 4.5 ± 0.6 for task trainer and verbal assessment techniques, respectively, a statistically significant difference (t = 2.3, df = 16, p = 0.034). The overall global rating scores for lumbar puncture task trainer and verbal assessment were 4.9 ± 0.2 and 4.8 ± 0.4, respectively, but were not statistically significant (t = 1.7, df = 16, p = 0.104).

For the central line procedure, differences in experience (number of years in practice) resulted in statistically significant checklist completion rates. The zero to five years in practice group had on average 12.8% higher percentage checklist score than participants with more than 10 years in practice. The zero to five years in practice group also had the largest difference (13.4%) between central line task trainer and verbal assessment format. Both males and females had similar significant improvements between task trainer and verbal assessment for percentage checklist items observed. Similar differences were observed between lumbar puncture task trainer and verbal assessments, and there were no significant differences for gender or experience.

Though there was discrepancy between the percentage checklist items observed score for central line verbal assessment technique (68%) and overall global assessment (90.6% (i.e., 4.53/5)), these two variables were moderate to highly positively correlated (Table 2). Similar results were observed for central line task trainer, though lumbar puncture procedure performance was not correlated.

**Table 2:** Correlation results of procedure, assessment technique, and evaluation format.

Procedure	Assessment technique	Variable 1	Variable 2	Correlation coefficient
Central line	Task trainer	Percentage checklist items observed	Overall global assessment	0.642
	Verbal	Percentage checklist items observed	Overall global assessment	0.644
Lumbar puncture	Task trainer	Percentage checklist items observed	Overall global assessment	-0.005
	Verbal	Percentage checklist items observed	Overall global assessment	0.375
Overall global assessment		Lumbar puncture - verbal	Lumbar puncture - task trainer	0.605
		Central line – verbal	Central line - task trainer	0.661

Of the survey respondents to the post-session survey (n = 17), 82% reported “strongly favoring” the simulation format for assessment over verbal format. More participants reported a strong preference for the simulated format for learning procedural skills (76.5%) than for assessment of procedural skills (64.7%).

Participants were surveyed regarding their confidence in performing procedures. Participants rating confidence in their central line skills as “very confident” increased from 71% prior to the session to 86% following the session. For lumbar puncture skills, “very confident” ratings increased from 57% prior to the session to 71% following the session.

## Discussion

The scores on the verbal assessment were significantly lower than those on the task trainer assessment for both central line placement and lumbar puncture. This supports the hypothesis that the verbal assessment and task trainer assessments would not yield similar results despite assessing the same procedure. One explanation for this discrepancy is that that the absence of kinesthetic triggers when verbally describing a procedure may have resulted in participants inadvertently overlooking a critical step. However, the physical act of performing the procedure allowed the participants to experience kinesthetic triggers to perform important steps, such as viewing the marks on the guidewire as a prompt for how far to insert the wire.

As an evaluation tool, lower scores on the verbal assessment are concerning because individuals who could perform well on a task trainer may have inadvertent consequences based on their verbal account of the procedure. In addition, lower scores on procedural checklists via verbal description seen in this study raise the concern that physicians with excellent procedural skills may be at a disadvantage if their procedural skills are assessed by oral examination. The preference expressed by participants for the task trainer-based assessment and the trend towards an increase in procedural confidence seen following this assessment imply a perceived benefit of the kinesthetic experience offered in the task trainer format.

This study is novel in its comparison of two possible forms of assessment. Medical specialty certification boards like the ABEM [3] and the American Board of Surgery [26] use oral examinations to determine board certification, prompting the selection of oral assessment as the control condition in this study. The Model of the Clinical Practice of Emergency Medicine lists 65 distinct procedures that are “integral to the practice of emergency medicine,” and the only means by which the Board currently examines these procedures are via written and oral examination. This study presents an alternative approach to assess procedural performance in attending emergency physicians. As assessment of physicians in practice becomes more common and recognized as a necessity, evaluation of assessment methods becomes increasingly important to ensure their accuracy and reliability. A natural progression for certifying boards with the increasing availability of task trainers with procedural fidelity could be to consider incorporation of those procedures into the process of board certification. This would require the development and validation of checklists for procedures that incorporate procedural variability among physicians while placing emphasis on steps integral to patient safety, such as maintaining a sterile field. In addition to the task trainer assessment option, future investigations could consider the use of a diversely functional virtual reality-based simulation as a versatile way to provide both kinesthetic cues and conserve resources, such as the cost of specific task trainers or procedural supplies.

The performance of attending emergency physicians was varied, but few reached the “minimum passing standard” of three or less missed items. A similar low passing rate was also observed in attending physicians of a broad range of specialties when assessed on the same checklist for central line in another study [20]. In our study, all subjects had the opportunity to practice their skills for improvement and perform a return demonstration of the missed steps, immediately following performance review. As the study was part of the OPPE, this allowed assessors to ensure that each physician was able to accurately demonstrate proficiency prior to completing the module. Future work could consider re-evaluation of the procedural checklist following this coaching period; however, recruitment for such a study may be limited by the added time required.

When expert raters provided a global assessment of performance, their ratings were higher relative to the scores on the checklist for both procedures and formats. These scores were positively correlated with checklist performance, but this does not explain the relatively higher score on a global rating. The score discrepancy could relate to bias on the part of the rater or could imply that the rater felt the missed items on the checklist were not as important in the safe performance of the procedure, thereby perceiving a higher level of skill than is possible to observe with the checklist. A contributor to bias may be familiarity of the rater with the physician participant from personal interactions in the clinical environment. The correlation between global rating score and checklist score is also seen in other studies of central line checklist adherence [24].

An observed difference among participants in this study is the decrease in checklist score seen with increasing years in practice. This was also demonstrated in another study examining physicians of many specialties [20,27]. This suggests that physicians with more years of experience do not have more procedural skill but in fact may have had skill decay, an outcome seen in other studies on advanced cardiovascular life support protocol adherence over time [2]. In addition, skills that are newer to emergency physicians’ skill set such as ultrasound guidance for central lines have to be mastered on a continuing basis by each attending in practice. However, there are no ongoing procedural practice requirements in emergency medicine at this time. This underscores the need for regular intervals of procedural practice for attending emergency physicians.

Limitations

This study had small attending physician sample size and single institution sampling. This research was performed in a state-of-the-art simulation lab with high-quality task trainers and trained faculty. There were eight expert raters, and this may have resulted in lower interrater reliability or confounding of data. Raters may have been positively or negatively biased by previous personal or clinical interactions with the physicians in the study. The second rater for each subject was limited by the quality of the recorded video and audio to confirm completion of checklist steps. Only two procedures were assessed using one checklist for each, and both procedures are commonly performed, so this assessment may not apply to rare procedures.

Further research is needed with a larger sample size or multi-institutional subject group to determine the reliability and validity of checklists for use in attending physicians. The variability in skill level that results from years of independent practice may result in lower compliance with certain elements of the checklist that may not directly affect patient safety. The development of separate initial competency and continuing training checklists may be appropriate to limit the effects of practice variations that are not known or suspected to have significant patient safety implications (for example, priming the central line tubing prior to placing the line). Currently, literature supports decreased central line-associated blood stream infections with implementation of a curriculum for residents [5], but it is not known which elements of the checklist resulted in this improvement. Another consideration is the use of a checklist developed using an evidence-supported method such as the Delphi method [28]. Finally, a checklist weighted for relative importance of certain skills should be considered.

## Conclusions

The average scores on procedural checklists were higher for simulation-based than for verbal assessment controls for both the central line placement and lumbar puncture procedures. Therefore, it can be suggested that the simulation format allows observation and evaluation of a larger number of procedural steps. Subjective ratings by global rating scale were better for those who performed well on the checklists for both procedures, suggesting that the checklists may be a good way to assess overall procedural skill. The simulated format was received well by subjects and was preferred to verbal assessment. Subjects had increased confidence in their procedural skills following completion of the session.

Evaluation of procedural skills over the course of an emergency medicine physician’s career can be considered valuable for both the kinesthetic reminder of rarely performed procedures and the potential for improved patient safety related to adhering to procedural guidelines. Consideration of task trainer-based simulated procedural assessment as an alternative to oral examination with verbal description may benefit candidates by more accurately demonstrating their procedural skill.
